# 

**Published:** 1892-01-02

**Authors:** 


A MAGISTRATE'S OPINION.—To the Editor of the "Daily Post."—Sir,—On Thursday I had a smart touch of Influenza, and have achieved such a rapid cure that it may be of service to your readers to know the means used. On Thursday I was totally incapacitated, on Friday convalescent, on Saturday quite well. Treatment each day—four dessert spoonfuls Cod-Liver Oil, one after each meal; four Teacups full of **BOVRIL** mid-way between meals, and last thing at night. I believe this will cure any one.—Yours, &c., (Signed) George S. Hazlehurst, The Grange, Rock Ferry. J.P., Flintshire.

### THE HOSPITAL

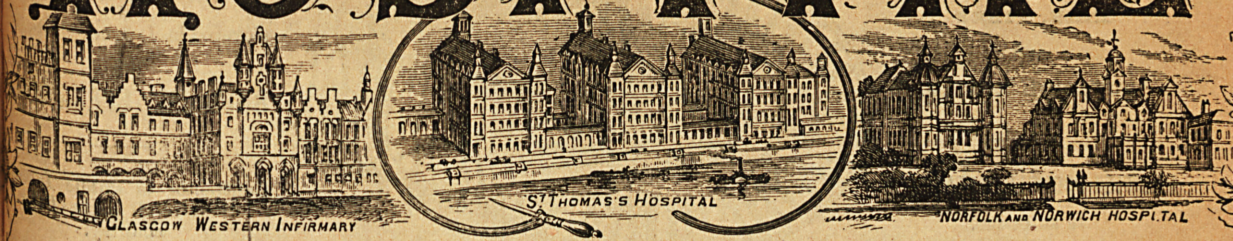

WITH EXTRA NURSING SUPPLEMENT.

No. 275.—Vol. XI.] SATURDAY, JANUARY 2nd, 1892. [ONE PENNY

